# Experimental Study on the Quenching Behavior of a Copper Cube in the Cellulose Nanofiber Solution

**DOI:** 10.3390/nano12061033

**Published:** 2022-03-21

**Authors:** Hundong Choi, Subin Jeong, Kwon-Yeong Lee

**Affiliations:** Department of Mechanical and Contol Engineering, Handong Global University, 558 Handong-Ro, Pohang-si 37554, Korea; hundongchoi@gmail.com (H.C.); sooibing@postech.ac.kr (S.J.)

**Keywords:** quenching, cellulose nanofiber, oxidization layer, gelation

## Abstract

This study investigates the quenching behavior and heat transfer characteristics of a copper cube immersed in cellulose nanofiber (CNF) solutions. The heat transfer performance of CNF solutions during boiling has been examined in several studies, but the quenching behavior of CNF solutions, which is an important heat transfer method, has not been evaluated. In this study, four copper cubes with the same dimensions and four different quenchants (deionized water and 0.01%, 0.1% and 0.5% CNF solutions) were prepared. A copper cube heated to greater than 600 °C was submerged three times in the quenchant. This was repeated with different copper cubes in each of the quenchants. The time at which the heated copper cube convectively transferred heat to the surroundings was recorded. The cooling time was shortest when the cube was immersed in a 0.5% CNF solution for the first time. The average cooling time for quenching in the 0.5% CNF solution was 30.3% shorter than that in DI water. In this study, film boiling during quenching was thoroughly observed and analyzed to explain the cause of enhancement in heat transfer efficiency.

## 1. Introduction

Quenching is a process in which rapid cooling occurs when a hot object is immersed in a coolant. It is observed both in nature and industry. When a volcano erupts under the sea, the lava is quenched by the surrounding water. Heated metals are frequently immersed in a coolant for many applications, such as the hardening of heated steel and rocket engines [1]. Quenching is also an important process for the safety of nuclear plants, especially when a severe accident such as the loss of coolant occurs [2].

Several studies on improving the heat transfer characteristics of quenching have been conducted focusing on using nanofluids as a coolant, altering the roughness of a metal surface through chemical treatment, or oxidizing metal surfaces [3,4,5,6,7]. Park et al. [3] first used alumina nanofluids to investigate the effect of nanoparticles on the film boiling heat transfer by comparing the results with those obtained in pure water. A sphere with a diameter of 10 mm was heated to a high temperature (1000–1400 K) and immersed in nanofluids with volume concentrations of 5% to 20%. Almost identical results were obtained with all of the nanofluid concentrations. Higher concentrations did not produce an enhancement in the heat transfer rate. Although the results showed that both the film boiling heat flux and heat transfer coefficient in nanofluids were lower than those in pure water, repeated quenching in nanofluids produced no film boiling. It was suggested that the film formation was disrupted by nanoparticle deposition on the quenched sphere. Kim et al. [4] quenched steel and zircaloy spheres using alumina, silica, and diamond nanoparticles at low concentrations (≤0.1 vol%). Quenching and boiling curves were obtained for different nanoparticles and the critical heat flux (CHF) and minimum heat flux (MHF) were compared with those of pure water. The CHF and MHF increased with repeated quenching when alumina and silica nanofluids were used, whereas the diamond nanoparticles did not result in a significant change. The authors suggested that the CHF and MHF were affected by the surface roughness and wettability due to nanoparticle deposition. Khoshmehr et al. [6] and Bolukbasi et al. [7] also investigated the quenching characteristics of carbon nanotube water and silica through comparisons with pure water. Both studies reported a reduced quenching time with repeated quenching and concluded that a change in the roughness of the surface due to nanoparticle deposition accelerated the quenching. Based on these previous results, nanoparticle deposition occurs during quenching and alters the surface characteristics, which is an important factor affecting the CHF and MHF of the fluid.

The surface roughness can be modified by oxidation. Lee et al. [8] conducted quenching experiments with vertical stainless steel and copper rodlets in pure water. During the experiment, both rodlets were heated under severe conditions for 2 h, cooled, and then quenched. The steel rodlet was not oxidized, whereas the copper rodlet showed a feather-like structure. Oxidation significantly changed the surface roughness of the copper rodlet. The quenching time with the copper rodlet having a feather-like structure decreased significantly. In addition, the rapid collapse of film vapor was observed with the 2-h oxidized copper rodlet as a result of the disruption of the film boiling caused by breaking the oxidized surface.

Meanwhile, the aggregation and sedimentation of nanofluids hinder their commercial use despite their CHF enhancement performance. The van der Waals forces and surface charges in water accelerate the aggregation and creation of clusters [9]. Moreover, it has been reported that the low dispersion stability of inorganic nanoparticles in solution reduces the thermal conductivity of the nanofluids and hence mitigates the boiling performance of the solution [10]. However, it is difficult to overcome the challenges of inorganic nanoparticles arising from their complex preparation and dispersibility in water. Hwang et al. [10] used cellulose nanofiber (CNF) as a solute to prepare nanofluids and studied the heat transfer characteristics of the solutions. The CNFs were dissolved with high stability, unlike other inorganic nanoparticles, owing to their high zeta potential. Moreover, the critical heat flux of CNFs was enhanced by 69.4% compared to that of pure water.

Choi et al. [11,12] also verified CNF as a possible working fluid using a two-phase closed thermosyphon (TPCT). They designed a closed copper pipe and heated it until the working fluid inside the pipe boiled. The heat transfer characteristics of the CNFs were investigated by comparing them with deionized (DI) water. The CHF and heat transfer coefficient of the system increased by at least 14.3% and 71.74%, respectively, when CNF was used as the working fluid. Furthermore, no sediment or aggregation was observed after the experiments. This further suggests that the nanoparticles can enhance the heat transfer characteristics without aggregation or sedimentation. Several studies on boiling CNF have been conducted and confirmed that the heat transfer characteristics of the system are increased when CNF is used as the working fluid. However, to the authors’ knowledge, there has been no research conducted on the heat transfer characteristics of CNF during quenching. Furthermore, heat transfer through boiling creates the possible danger of overheating. An overheated system must be cooled instantly to avoid system failure and catastrophic disasters. Therefore, in this study, the quenching behavior of CNF nanofluids are further investigated.

## 2. Experimental Approach

**Figure 1:**
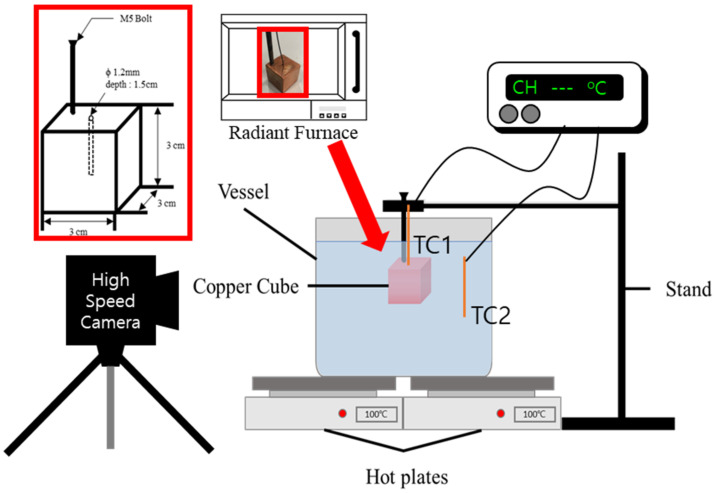
Schematic diagram of experimental setup.

The experimental apparatus for the present study consists of the tested cubic copper, a radiant furnace (LABOTEC, SH-FU-14MG, Seoul, Korea), a clamp and stand, a quench pool, a data acquisition system (Keysight Korea, 34970A, Seoul, Korea), and a high-speed camera (Optronis, CR-S3500, Nikon Sigma 105 mm, Kehl, Germany) as shown in Figure 1. The well-polished cubic copper specimens have dimensions of 30 mm × 30 mm × 30 mm. A threaded hole with a depth of 20 mm is drilled at the edge of each specimen and tightened with a screw of M5 × 100 mm (Celbit, SUS304, Pohang, Korea). In another hole drilled to a depth of 15 mm at the center of the cube, a K-type thermocouple (TC1) is inserted and attached with ceramic glue to ensure good thermal contact. 

Owing to the high thermal conductivity of the material, it is assumed that the temperatures inside and on the outside surface of the specimen are ideal. Therefore, the temperature of the center is measured, rather than that at the surface of the specimen. The heat flux of the copper cube is calculated using the lumped capacitance method, as given in Equation (1) [13]:

(1) $$q^{\prime \prime }=-h_{f}\left(\Delta T\right)=-\frac{\rho cV}{A}\frac{d\Delta T}{dt}$$

where $q^{\prime \prime }$, ρ, and c are the heat flux, density, and heat capacity of the specimen, respectively; *V* and *A* are the total volume and area of the specimen, respectively; and dΔT/dt is the rate of temperature change during the time interval [13]. The dimensionless Biot number must be calculated and considered for the lumped capacitance method. The equation for the Biot number is given in Equation (2) [13]:

(2) $$Bi=\frac{h_{f}L_{c}}{k_{c}}$$

where Bi is the Biot number, $h_{f}$ is the heat transfer coefficient during film boiling obtained from Equation (1), L is the characteristic length of the specimen (the volume of the specimen divided by the area of the specimen), and $k_{c}$ is the thermal conductivity of copper at 873 K. When the Biot number is less than 0.1, the heat flux of the specimen can be evaluated with an error of less than 0.5% [13]. The maximum calculated Biot number of the system was 0.012 during film boiling. ($h_{f}=4.21\times 10^{5}\;W/m^{2}\cdot K$, $L_{c}=10^{-5}\;m$, $k_{c}=361\;W/m\cdot K)$ Hence, the surface temperature of the specimen was estimated using the lumped capacitance method.

The heated copper cube was inserted slowly using stands with clamps. A vessel with a size of 275 × 200 × 135 mm was filled with DI water and CNF solutions with concentrations of 0.01%, 0.1% and 0.5%. These discretized domains are obtained from the previous researches where significant differences during heat transfer were found due to the different weight percentage of the CNF solution [10,11,12]. The pool temperature was maintained by two hot plates below the vessel and monitored using a K-type thermocouple (TC2). To validate the repeatability of the test, each specimen was quenched three times in the same solution.

Commercial CNF (2 wt%), an eco-friendly, lightweight, non organic nanofiber was purchased from ANPOLY (Pohang, Korea) and used as a solute. It was produced using a TEMPO-mediated oxidation method [14]. The length of individual fiber should be a few microns and a width of 5–10 nm. Since CNF was supplied in the form of a gel with a concentration of 2%, sufficient ultra-sonification was conducted after mixing with DI water until the solution became transparent. CNF solutions with different concentrations were prepared after complete dissolution.

The test cube was heated in a radiant furnace at 4500 W (220 V, 20.5 A). The temperature was measured using a K-type thermocouple and monitored by a data acquisition system. The temperature of the quenchant was maintained at a saturation temperature of approximately 100 °C using two hot plates. The cube was withdrawn from the furnace when the temperature reached 800 °C. The heated cube was placed and hung using the stand and slowly immersed into the pool when the temperature was above 600 °C. During immersion, the boiling phenomenon was filmed using a high-speed camera. During the experiment, the temperature of the cube was continuously recorded at a rate of 20 Hz to obtain the quenching curve.

All of the measurements and recordings ended when the temperature of the cube reached a saturated temperature, at which point the cube was removed from the quenchant. The experiment was repeated with the same specimen and quenchant three times.

## 3. Results and Discussion

### 3.1. Effect of Oxidation Layer during Quenching in DI Water

**Figure 2:**
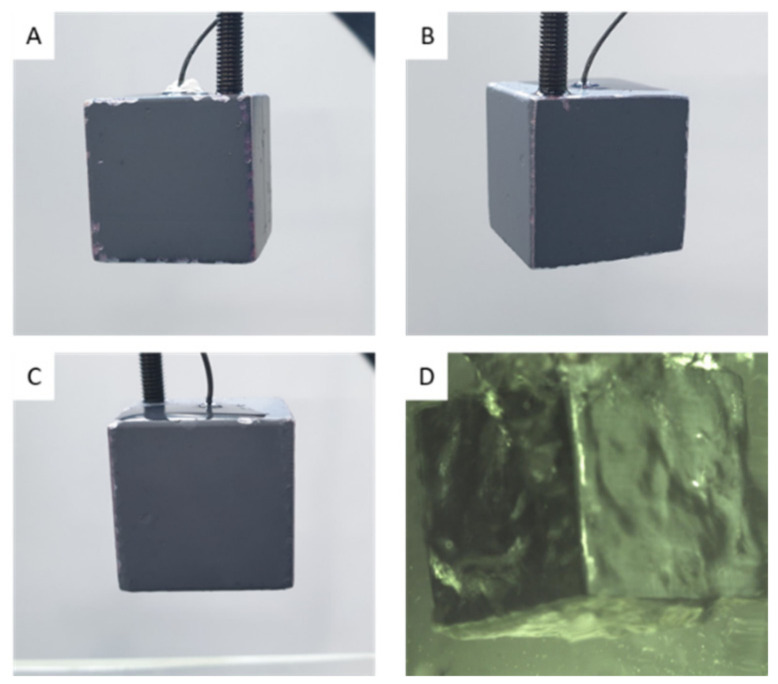
(**A**) After 1st Quenching with a copper cube, (**B**) After 2nd quenching (**C**) After 3rd quenching (**D**) Copper cube immersed in water during first quenching.

At high temperatures, copper reacts with oxygen to form copper oxide (CuO). The surfaces of the copper cube turned black during heating in the furnace due to oxidation. From the first to third quenchings in DI water, the surface of the copper cube remained black, and the second and third experiments exhibited almost the same quenching behavior. Although nucleate boiling occurred vigorously when the cube reached the saturation temperature (100 °C), the oxidation layer was still observed after quenching, as shown in Figure 2A–C. Figure 2D shows the copper cube during quenching in DI water; the oxidation layer formed during the heating process did not break off during the entire quenching process.

**Figure 3:**
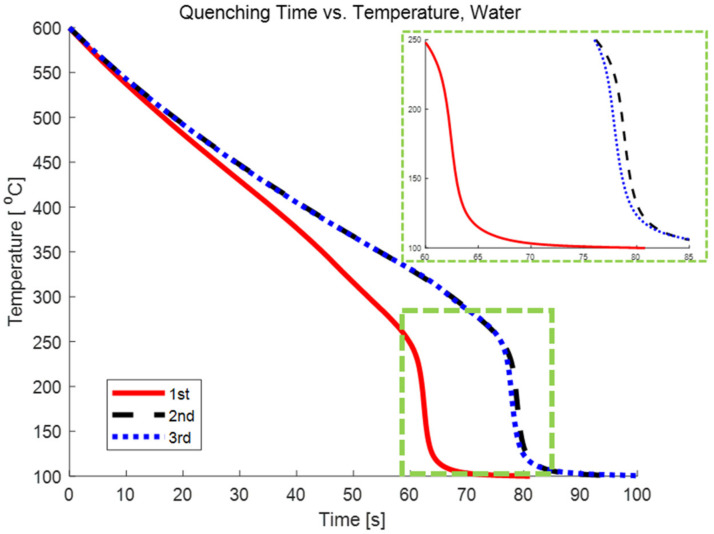
Quenching curves of a copper cube in DI water.

The oxidized surfaces and repeated experiments with the same cube hindered the heat transfer characteristics, as shown in Figure 3. The typical thermal conductivities of CuO and Cu are 33 W/mK and 400 W/mK, respectively [15]. The CuO layer with low conductivity on the copper cube acts as an insulator and prevents heat from escaping. With repeated experiments, the oxidized layer was continuously deposited on the surface until no further oxidized layers could be deposited. The oxidized layer on the copper cube was saturated, and therefore similar quenching times were obtained for the second and third experiments.

### 3.2. Quenching Behavior with Increasing Concentration of CNF Solution

**Figure 4:**
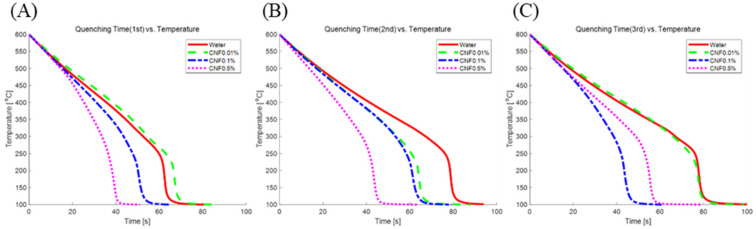
Cooling curves in different quenchant; DI water, CNF 0.01%, CNF 0.1%, and CNF 0.5% (**A**) First (**B**) Second (**C**) Third Quenching.

Figure 4 shows the cooling curves for specimens quenched three times in four different quenchants: DI water, 0.01% CNF, 0.1% CNF and 0.5% CNF. The cooling curves in Figure 4 show that the quenching in each solution behaved differently, especially during film boiling. The cooling times in Table 1 represent the time at which the temperature of the specimen reached 100 °C. When the specimens were quenched for the first and second times, the high concentration of CNF cooled the hot copper cube faster, while the copper cube quenched in 0.1% CNF solution cooled faster than the other three cases during the third quenching. However, when comparing the first quenching in all solutions, the hot copper cube cooled 36.5% faster in the 0.5% CNF solution than in DI water. Even when comparing the averaged quenching time, the 0.5% CNF solution showed the fastest average cooling time (30.3% faster than DI water), which means that the slope of the cooling curve for the 0.5% CNF solution is steeper than that of the other three solutions during film boiling, as shown in Figure 4.

**Figure 5:**
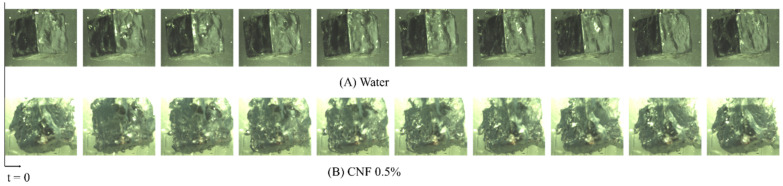
Photographs of the film boiling during first quenching in (**A**) Water (**B**) CNF 0.5% solution. The interval between frames is 5 ms.

Since CNF solution is transparent as stated above, it is possible to clearly observe the bubble dynamics along the surface of copper cubes. Figure 5A,B show photographs taken by the high-speed camera with a time interval of 5 ms. The vapor blanket behaviors around the copper cube during quenching in water and the 0.5% CNF solution during film boiling are clearly different. When the heated copper cube was immersed in DI water, it was surrounded by a complete vapor blanket, which prevented direct contact between the solid and quenchant (Figure 5A), whereas the vapor blanket was unstable in the 0.5% CNF solution (Figure 5B). The unstable film boiling can be explained by the high zeta potential of CNF (−96 mV) and nanoparticle deposition on the surface.

**Figure 6:**
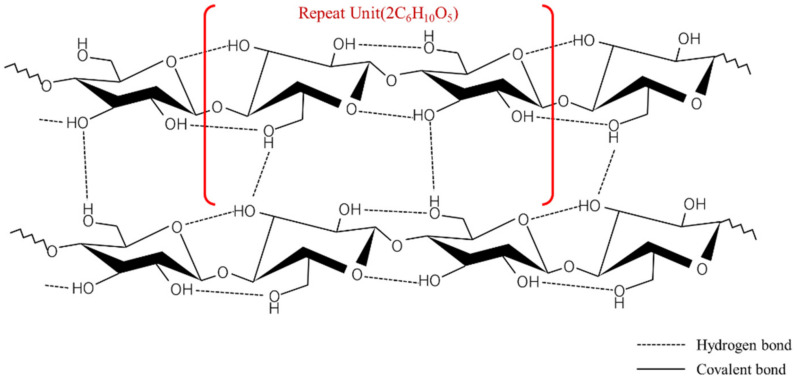
Atomic structure of CNF with repeating anydro-glucose rings.

**Figure 7:**
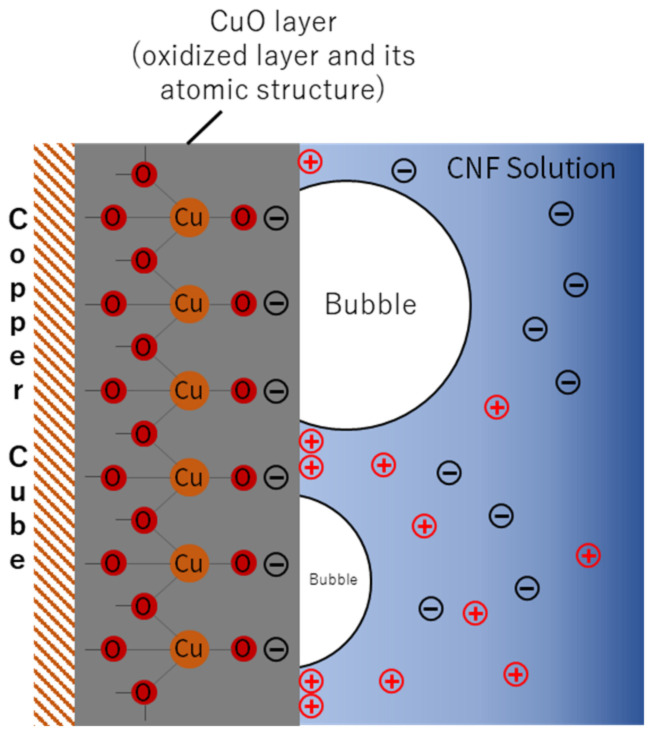
Ions represented around a cupper cube during the quenching.

Zeta-potential of solute describes the colloidal stability in the solution. According to Hsu et al. [16], a high zeta potential tends to initiate an electrical double layer and disturb the vapor blanket formation. They investigated the quenching behavior in NaCl solution (seawater) and suggested that the relatively high zeta potential of seawater (from −7 mV to −10 mV) hindered film boiling and accelerated quenching. The CNF solution has a zeta potential more than nine times higher than that of seawater. This is induced by covalent bonding between two anhydro-glucose rings (C_6_H_10_O_5_) and hydrogen bonding in and between repeated units of anhydro-glucose rings, as shown in Figure 6 [17]. When CNFs are dissolved in DI water, they also form hydrogen bonds with the DI water and exist as positive and negative ions. The positive ions in the solution are attracted to the copper oxide layer, while the negative ions are repelled, as shown in Figure 7. The positive ions attached to the copper oxide layer block the vapor blanket formation, particularly during film boiling. Although the zeta-potential does not change with the concentrations owing to its definition, the ions present in the solution will be much less when the concentration is low. Number of ions presented in the solution affected the heat transfer between the solution and specimen, caused the dramatic slope changes of the 0.5% CNF solution (Figure 4) and accelerated the quenching while quenching in 0.01% CNF solution showed the similar behavior with the DI water. The higher the concentration of CNF solution, the more improved heat transfer efficiency, while other nanometal nanofluids did not guarantee heat transfer enhancement at higher concentration.

**Figure 8:**
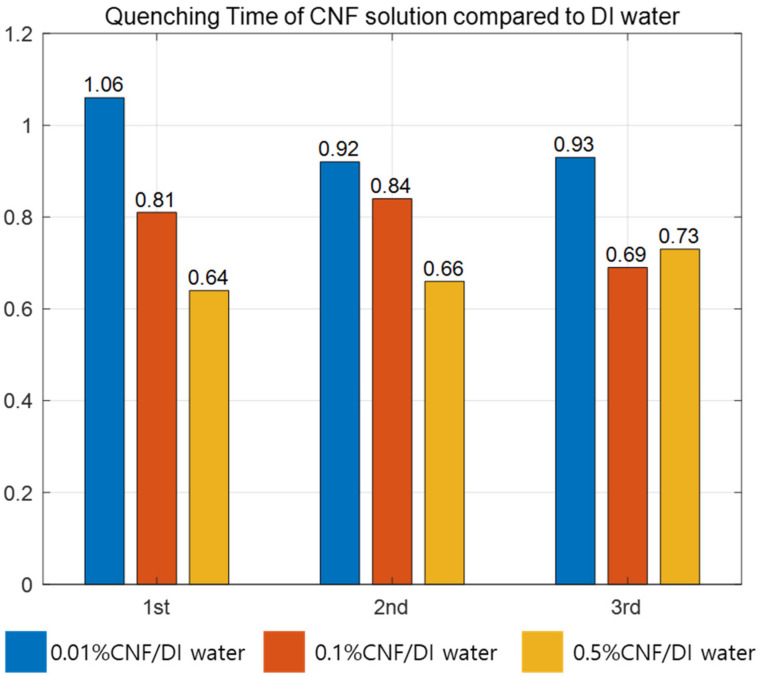
Cooling time of CNF solution compared to DI water.

It is well-known fact that the nanoparticle deposition increases wettability and heat resistance ($R_{nano}$) at the same time. As shown in Table 1, the average times taken for quenching in DI water and CNF 0.01% did not show much difference. Moreover, the 1st trial case, DI water showed better quenching performance than CNF 0.01%. (Figure 8) According to the effect of nano-particle deposition on the heat transfer, breaking film on the surface of the specimen should have led to heat transfer enhancement. However, nano-particles did not deposit on the surface enough with the low density of CNF to generate the wicking force, but became another source of heat resistance. Eventually, low density of CNF (0.01%) does not effectively enhance heat transfer but even showed lower performance in first trial compared to DI water. The continuous quenching in 0.01% CNF solution consequently increased the deposition of nano-particle on the specimen and showed better heat transfer performance at the end. The sudden decline of quenching time in 0.1% CNF could also be interpreted by the addition of the nano-particle deposition on the copper surface as the quenching trial increases (Figure 8).

### 3.3. Gelation and Damages on Oxidation Layer during Quenching

**Figure 9:**
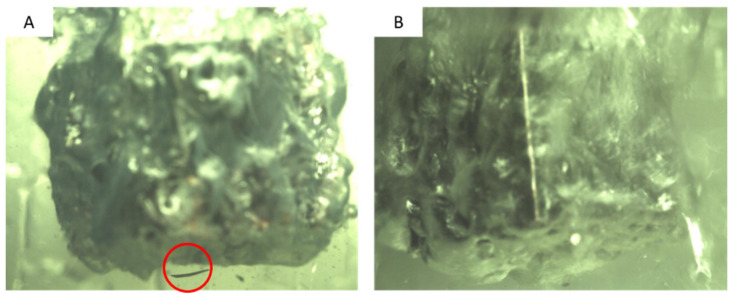
Copper cube (**A**) quenching in CNF 0.5% during film boiling (**B**) quenching in DI water during transient boiling.

Surface roughness and modification can significantly affect the film boiling heat transfer. Lee et al. [8] found that the oxidation layer works as another heat resistance, and thus increasing cooling time. As shown in Figure 9A,B, the oxidized layer was removed during film boiling in 0.5% CNF solution, while it remained in place even during the vigorous transient boiling in water. The broken oxidized layer is indicated by the red circle in Figure 9A.

Woo et al. [18] suggested that the carboxylate content in CNF reacts with copper (II) ions to form stable CNF-Cu. The reaction between CNF and Cu occurs at room temperature and is accompanied by gelation. Bonding between CNF and copper (II) suggests that the reaction below is likely to occur when the copper cube is immersed in the CNF solution.

(3) $$4RCOO^{-}+2CuO\to 2{\left[RCOO\right]}_{2}Cu+O_{2}$$

**Figure 10:**
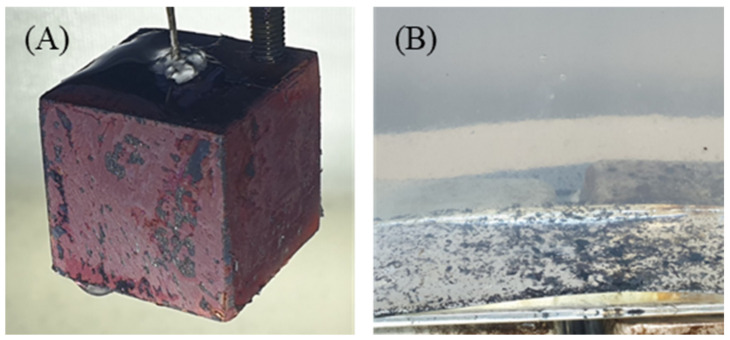
(**A**) Copper cube surface after the first quenching in 0.5% CNF solution (**B**) CNF-Cu aerosol sediment after the third quenching in 0.5% CNF solution.

**Figure 11:**
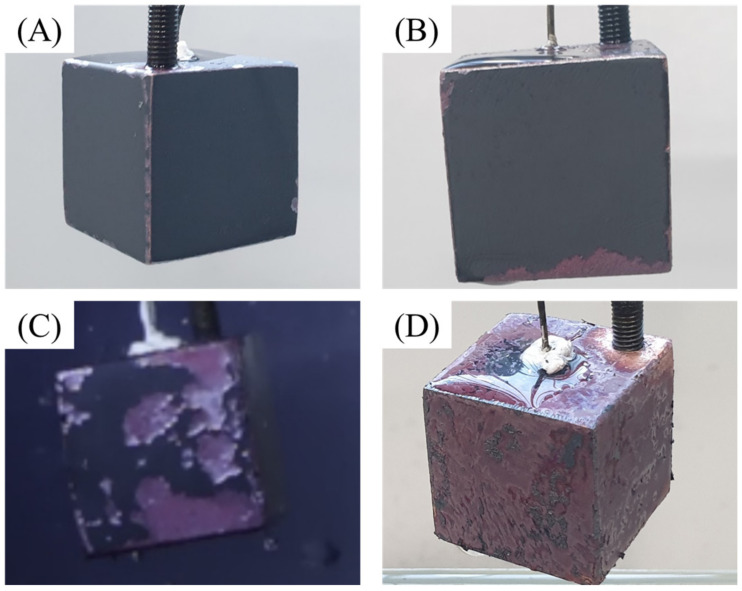
Copper cube surface after the first quenching in (**A**) DI water, (**B**) 0.01% CNF solution, (**C**) 0.1% CNF solution, and (**D**) 0.5% CNF solution.

**Figure 12:**
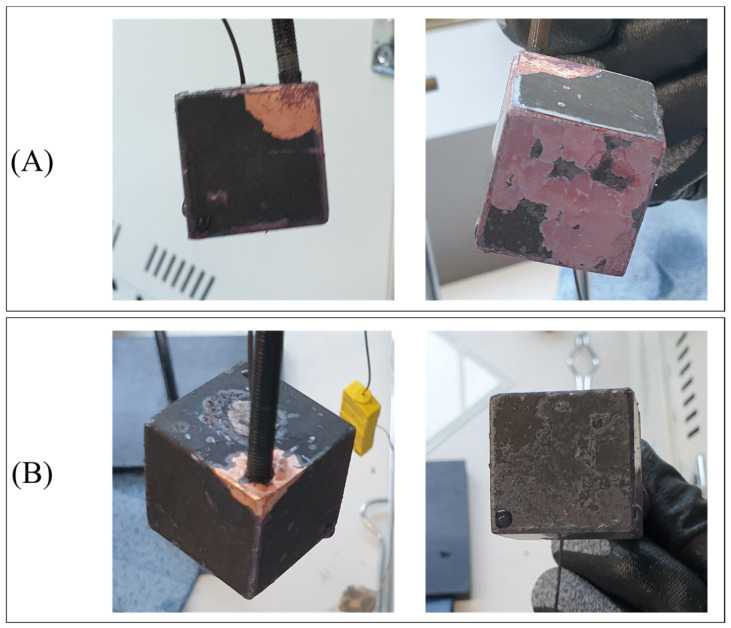
The copper cube after (**A**) first quenching in 0.1% solution, (**B**) the last quenching in 0.1% solution.

The reaction between the carboxylic content and the copper cube is demonstrated by the gelation after quenching, as shown in Figure 10. The glittering surface shown in Figure 10A indicates that gelation occurred during quenching and covered the copper surface. Figure 10B illustrates the gelation sediment after the first quenching in the 0.5% CNF solution. As mentioned previously, the unstable film boiling layer caused by the high zeta potential enabled direct contact between the oxidized layer and CNF solution when the copper cube was still hot. The reaction tended to occur during both film and transient boiling. As the concentration increased, the carboxylic content of the solution also increased. Solutions with a higher concentration of CNF tended to react with the copper cube more extensively, breaking the copper oxide. Figure 11 shows the damaged oxide layers of the four copper cubes after the first quenching in four different solutions. As the concentration increased, the contact between the copper oxide and CNF solution at high temperatures increased as a result of unstable film boiling, and the layer broke more vigorously. The broken layer during film boiling disturbed the vapor blanket formation around the cube and accelerated the heat transfer between the specimen and solution. Figure 12A) shows the image of the specimen after the first quenching, which shows the severe break of oxidized layer at the bottom of the specimen. On the other hand, Figure 12B) depicts the almost undamaged oxidized layer after third quenching. During first quenching in 0.1% solution, the breakage of oxidized layer played a significant role in decreasing the quenching time, while the dense nano-particle deposition increased the heat transfer resistance in third quenching in 0.5% solution as stated above.

## 4. Conclusions

All overheated systems sometimes undergo quenching processes, especially in emergencies. The heat transfer characteristics of CNF solutions have been investigated by other researchers. In this study, the quenching behavior of CNF solutions was tested and reviewed. The quenching time of a hot copper cube was reduced by a maximum of 36.5% when the cube was cooled in a 0.5% CNF solution. This is due to two effects: a double electrical layer around the cube formed as a result of the high zeta potential of the CNF solution, and the reaction between CNF and copper (II) oxide. This new organic nano-solution exhibited enhanced heat transfer performance and has the potential to be implemented in many other applications, such as cooling heated parts in nuclear power plants.

## Figures and Tables

**Table 1:** Cooling time in DI water, CNF 0.01%, CNF 0.1%, and CNF 0.5%.

Solution	Unit	1st	2nd	3rd	Average	vs. DI Water
DI water	[s]	81.0	94.2	104	93.1	-
CNF 0.01%	[s]	86.2	88.4	98.8	91.1	−2.0(2%)
CNF 0.1%	[s]	66.8	79.8	73.0	73.2	−24.6(26%)
CNF 0.5%	[s]	51.4	64.2	79.2	64.9	−28.2(30%)
